# Efficient co‐production of propionic acid and succinic acid by *Propionibacterium acidipropionici* using membrane separation coupled technology

**DOI:** 10.1002/elsc.202000103

**Published:** 2021-06-07

**Authors:** Xiaolian Li, Liquan Wei, Ziqiang Wang, Yunshan Wang, Zhiguo Su

**Affiliations:** ^1^ State Key Laboratory of Biochemical Engineering Institute of Process Engineering Chinese Academy of Sciences Beijing P. R. China

**Keywords:** combination of glucose and glycerol, membrane separation coupled fermentation, *Propionibacterium acidipropionici*, propionic acid, succinic acid

## Abstract

To improve the fermentation efficiency of *Propionibacterium acidipropionici*, a semi‐continuous coupled fermentation process was established to achieve co‐production of propionic acid (PA) and succinic acid (SA). First, the optimal proportion of glucose (Glc) and glycerol (Gl) as a mixed carbon source was determined, and the feeding procedure of Gl was optimized to make more energy flow in the direction of product synthesis. Then, ZGD630 anion exchange resin was used for efficient adsorption of PA, thereby eliminating the feedback inhibition effect of PA. Finally, an efficient, coupled fermentation process of *P. acidipropionici* characterized by membrane separation and chromatography technology was developed. The concentrations of PA and SA reached 62.22 ± 2.32 and 20.45 ± 1.34 g L^−1^, with corresponding productivity of 0.43 and 0.14 g L^−1^ h^−1^, increased by 65.38% and 48.54%, respectively. Membrane separation coupled fermentation of PA and SA could significantly improve the process economics of *P. acidipropionici*, and has good prospects for industrial application.

**Abbreviations**
Gl: glycerol
Glc: glucose
HAc: acetic acid
PA: propionic acid
SA: succinic acid

## 1 INTRODUCTION

Propionic acid (PA) and its salts (e.g., calcium propionate, zinc propionate, potassium propionate) can effectively inhibit molds, gram‐negative bacteria, *Bacillus aerobacter*, and are widely used in grain, feed and food processing as preservatives [1]. Generally, PA is produced from petroleum‐based chemicals, raising concerns about its long‐term sustainability. Therefore, the production of PA by microbial fermentation has attracted widespread attention [2]. In particular, bio‐produced PA or its calcium salt is considered a safe and pollution‐free natural antifungal agent for use in food, approved by the World Health Organization and the Food and Agriculture Organization of the United Nations [3].

Succinic acid (SA) is another important building block for deriving value‐added chemicals in industry. Bio‐based SA production from renewable raw materials is a feasible approach to partially alleviate the dependence of global manufacturing on petroleum [4]. Although the production of SA by *Actinobacillus succinogenes*, *Escherichia coli*, and *Saccharomyces cerevisiae* has become very competitive with great efforts made in strain modification and process optimization, further improvement in the economics of production is required for industrial applications [5, 6].

**FIGURE 1:**
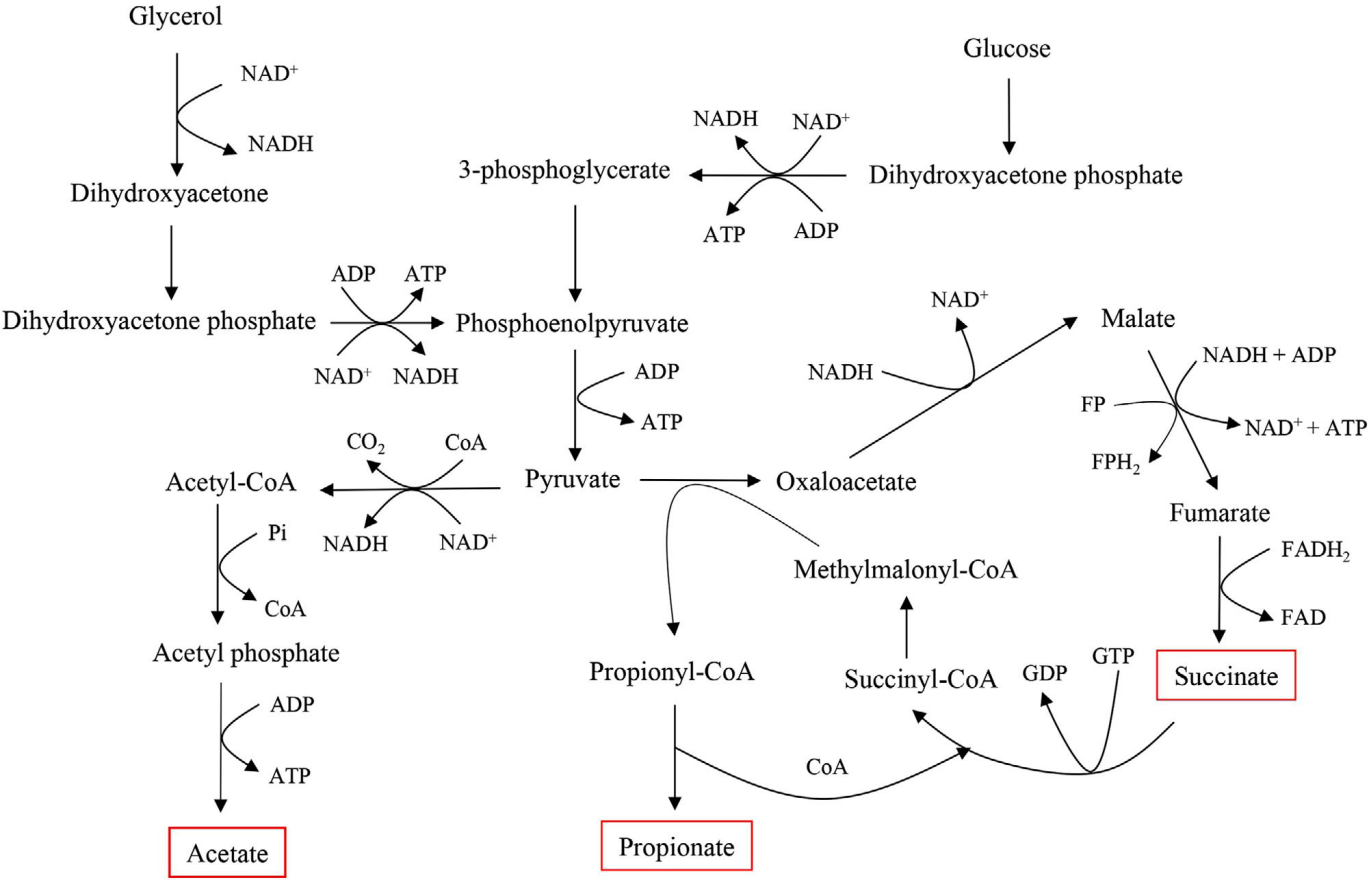
The metabolic pathway of PA in *Propionibacteria*

Propionibacteria are gram‐positive facultative anaerobic bacteria that have been granted “generally recognized as safe” status by the US Food and Drug Administration [7]. They are widely used in the production of PA by fermentation, such as by *Propionibacterium acidipropionici*, *P. freudenreichii*, and *P. shermanii*. PA fermentation is known to suffer from end‐product inhibition and byproduct formation, mainly SA and acetic acid (HAc), which lower the yield and productivity of PA [8]. Considerable efforts have been made to improve the fermentation efficiency. Some studies used metabolic engineering to enhance the producer strains, which has proven to be difficult [9, 10, 11], while others focused on medium composition [Co^2+^, corn steep liquor, and glycerol (Gl)] and process optimization [12, 13]. Among these methods, in situ product removal (ISPR) is a useful technique—it removes PA in time to effectively overcome its feedback inhibition effect, and helps to realize a semi‐continuous fermentation process. Various ISPR processes have been developed using an in‐situ cell retention reactor [14], plant fibrous‐bed bioreactor [15, 16, 17], or PEI‐Poraver immobilized bioreactor [18]. However, these studies only focused on a single product, and few works have been done on the applicability of co‐production of PA and SA. According to the metabolic pathway of PA in propionibacteria (Figure 1), the last step of propionate synthesis is the transfer of CoA from propionyl‐CoA to SA, indicating that the metabolism of PA and SA is inherently related [19, 20].

**PRACTICAL APPLICATION**

To improve the fermentation efficiency of *Propionibacterium acidipropionici*, first, the effects of glucose (Glc) and glycerol (Gl) on cell growth and product synthesis were analyzed; an optimal combination of Glc/Gl and feeding procedure of Gl were determined to make more energy flow in the direction of product (propionic acid [PA] and succinic acid [SA]) synthesis. Then, ZGD630 anion exchange resin was used for efficient adsorption of PA from the fermentation broth to eliminate the feedback inhibition effect of PA. Finally, semi‐continuous fermentation of *P. acidipropionici* characterized by the use of membrane separation and chromatography technology was developed to achieve co‐production of PA and SA. These developments significantly improve the process economics of *P. acidipropionici* and have good prospects for industrial application.

Another effective way to reduce the cost of PA fermentation is to identify a low‐cost, renewable feedstock. Gl, an abundant renewable byproduct of the biodiesel industry, has been used in the fermentation of *P. acidipropionici* [21]. Gl can promote the synthesis of PA and reduce the formation of byproducts. However, when used as the sole carbon and energy source, Gl slows down cell growth because of metabolic imbalance, so a combination of glucose (Glc) and Gl is required to maintain high productivity [22, 23].

In this paper, to improve the economics of *P. acidipropionici* fermentation, the effects of Glc and Gl on cell growth and product synthesis were analyzed, and then an adsorption process for PA in the fermentation broth was established. Finally, a semi‐continuous fermentation process of *P. acidipropionici* characterized by membrane separation and chromatography technology was developed to achieve co‐production of PA and SA.

## 2 MATERIALS AND METHODS

### 2.1 Microorganism and medium

*P. acidipropionici* CGMCC 1.2230 was obtained from the China General Microbiological Culture Collection Center. The stock culture was inoculated on an agar slant at 30°C, stored at 4°C, and transferred to a new agar slant monthly. Inoculum medium consisted of Glc (35 g L^−1^), corn steep liquor (21 g L^−1^), (NH_4_)_2_SO_4_ (5 g L^−1^), KH_2_PO_4_ (4 g L^−1^), and distilled water (pH 6.8–7.0). Fermentation medium was composed of Glc (60 g L^−1^), corn steep liquor (41 g L^−1^), KH_2_PO_4_ (4.6 g L^−1^), and distilled water (pH 6.8–7.0). For medium preparation, Glc was autoclaved separately.

### 2.2 Fermentation of *P. acidipropionici*

*P. acidipropionici* CGMCC 1.2230 on an agar slant was activated at 30°C for 24 h. One loopful of culture from the activated slant was inoculated aseptically into 50 mL of inoculum medium and cultivated statically at 30°C for 48 h. Then, this culture was inoculated into 500 mL of fresh inoculum medium and cultivated for 48 h. This culture was used to inoculate fermentation medium in a 10‐L fermenter at 70% capacity, operated at 30°C, 50 rpm. During the fermentation, no sterile air was required, and the pH was controlled at 6.5 by the automatic addition of NH_3_·H_2_O. Glc (500 g L^−1^) was fed to maintain cell growth and product synthesis when its concentration fell to <10 g L^−1^, with total Glc amount of 100 g L^−1^. The concentrations of Glc, PA, SA, and HAc were determined every 12 h.

### 2.3 Carbon source optimization and Gl feeding procedure

The fermentation process of *P. acidipropionici* was analyzed when Glc or Gl was used as the sole carbon source, with a total amount of 100 g L^−1^, respectively. Then the production of PA, SA, and HAc during the fermentation process was compared with different ratios of Glc to Gl as the carbon source. The total carbon source concentration was still 100 g L^−1^, and the ratios of Glc to Gl were 1:4, 2:3, 1:1, 3:2, and 4:1, respectively. Based on these experiments, the Gl feeding procedure was optimized to improve the fermentation efficiency.

### 2.4 Screening of ion‐exchange resin for PA removal

To improve the removal efficiency of PA, seven anion exchange resins were screened, including four strong‐base resins (ZGA302, ZGA304, ZGA351, D213) and three weak‐base resins (ZGA313, ZGA412, ZGD630). To measure the capacity of these resins, adsorption experiments were performed in the fermentation broth at pH 6.5. Pretreated resin (4 g) was added into a 100‐mL shaking flask containing 25 mL of fermentation broth, and reacted for 4 h at 30°C, 100 rpm. The adsorption capacity was determined by analyzing the PA concentration in the fermentation broth before and after adsorption with the following formula:

$$q_{e}=\frac{(c_{0}-c_{e})\times V}{W}$$

where *q*_e_ is the absorption capacity (mg g^−1^), *c*_0_ is the initial concentration (mg mL^−1^), *c*_e_ is the residual concentration (mg mL^−1^), *V* is the fermentation broth volume (mL), and *W* is the quantity of resin (g).

### 2.5 Semi‐continuous coupled fermentation of *P. acidipropionici*

**FIGURE 2:**
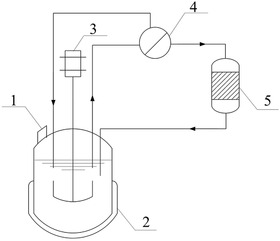
Schematic diagram of membrane separation coupled fermentation of *P. acidipropionici*. 1. Feed port/ Inoculation port; 2. Fermenter; 3. Motor; 4. Membrane device; 5. Chromatographic column. *P. acidipropionici* cells were separated on line with a membrane device when the PA concentration reached about 25–30 g L^−1^. The penetrant flowed through ZGD630 resin chromatography column to adsorb PA, and then returned to the fermenter for continuous fermentation

A membrane separation coupled fermentation process of *P. acidipropionici* was established according to Wang et al. [24]. During the fermentation process, a multifunctional membrane separation device equipped with a G13 plunger diaphragm pump (RNF 0460‐011), purchased from Xiamen Filter & Membrane Technology Co., Ltd. (China), was connected directly to the fermenter via a pipeline, and the fermentation broth was pumped circularly through a 0.22‐μm spiral membrane module (Type 1812) when the concentration of PA reached about 25–30 g L^−1^. The penetrant, containing Glc, PA, SA, HAc, et al., gradually permeated through the membrane module, and was then loaded onto a chromatography column packed with anion exchange resin. After adsorption of PA, the flow‐through liquid returned to the fermenter for continuous fermentation (Figure 2). To ensure aseptic operation, the membrane pipelines were cleaned with 0.5% w/w sodium bisulfite solution for 40 min.

### 2.6 Cell concentration

Cell growth was analyzed photometrically at 600 nm. Before measuring, *P. acidipropionici* cells were centrifuged at 10,000 × *g* for 10 min, and then resuspended in isovolumetric phosphate buffer (20 mM, pH 7.0) to eliminate the influence of corn steep liquor and other components.

### 2.7 Assay of organic acids and Glc

After centrifugation of the fermentation broth at 10,000 × *g* for 10 min, the supernatant was used to analyze the concentrations of organic acids and Glc.

High‐performance liquid chromatography (HPLC) analysis of PA, SA, and HAc was performed using a Shimadzu LC‐20AT HPLC system fitted with a Bio‐Rad HPX‐87H column (5 μm, 380 mm × 4.6 mm) and an SPD‐20A UV detector operated at 215 nm. The mobile phase was 5 mM H_2_SO_4_ with a flow‐rate of 0.6 mL min^−1^ at 55°C. The injection volume was 10 μL. Commercially available organic acids were used as external standards.

Residual Glc was determined enzymatically using a biological sensor, purchased from the Biology Institute of Shandong Academy of Sciences (Jinan, Shandong, China). The tested supernatant was diluted to a final Glc concentration in the range 0–1 g L^−1^, and then loaded onto the sensor with an injection volume of 25 μL. The Glc concentration was calculated according to a Glc standard solution.

### 2.8 Statistical analysis and yield and productivity calculation

All experiments were carried out at least in triplicate and mean and standard deviation (SD) values were calculated using the built‐in statistical tools for data analysis in Microsoft Excel. Productivity was calculated by dividing the concentration of organic acids at a certain time by the time elapsed at this stage, and is expressed in units g L^−1^ h^−1^. Yield was calculated by dividing the mass of organic acids produced by the total amount of Glc or/and Gl consumed at this stage, and is expressed in units g g^−1^.

## 3 RESULTS AND DISCUSSION

### 3.1 Fed‐batch fermentation of *P. acidipropionici*

**FIGURE 3:**
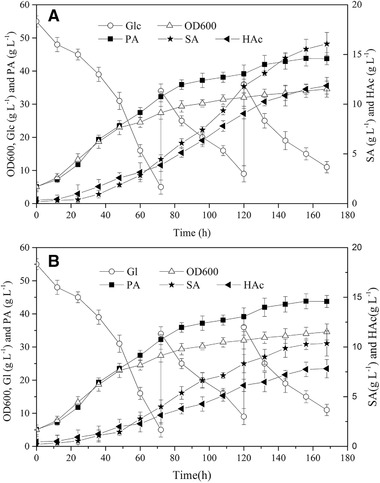
Time courses of OD600, Glc, Gl, PA, SA, and HAc in *P. acidipropionici* fermentation process. (A) Glc as the sole carbon source; (B) Gl as the sole carbon source

In the fermentation process of *P. acidipropionici*, the consumption of Glc and the production of organic acids, such as PA, SA and HAc, were analyzed. As shown in Figure 3A, cell growth was accompanied by the synthesis of organic acids with the consumption of Glc. When the Glc concentration was <10 g L^−1^, Glc (500 g L^−1^) was fed stepwise to maintain cell growth and product synthesis, with total Glc amount of 100 g L^−1^. It was reported that the productivity of PA slowed down when its concentration accumulated to about 25–30 g L^−1^ because of feedback inhibition [19, 25]. The results showed that the productivity of PA decreased after 84 h, and the corresponding PA concentration was 35.86 ± 1.36 g L^−1^. In the initial (0–84 h) and latter (84–168 h) stages of the fermentation process, the productivity of PA was 0.43 and 0.09 g L^−1^ h^−1^, respectively, with an overall productivity of 0.26 g L^−1^ h^−1^. At the end of the fermentation, the concentrations of PA, SA, and HAc were 43.74 ± 1.52, 16.07 ± 1.12, and 11.86 ± 0.84 g L^−1^, with a total amount of 71.67 g L^−1^. The corresponding yields were 0.44, 0.16, and 0.12 g g^−1^, respectively. Furthermore, different concentrations of PA were added into the fermentation medium to analyze its negative effect on cell growth. It was found that the rate of cell growth slowed down with the increase of the concentration of PA. Particularly, when the concentration of PA reached 30 g L^−1^, cell growth almost stopped [26, 27]. In the fed‐batch fermentation process, when the concentration of PA reached about 25 g L^−1^, the rate of cell growth slowed down obviously. Therefore, the threshold value of PA caused feedback inhibition was 25–30 g L^−1^.

### 3.2 Carbon source optimization and Gl feeding procedure

Gl could increase the production of PA and reduce the production of byproducts [22]. Therefore, the fermentation process was analyzed with Gl as the sole carbon source. As shown in Figure 3B, the concentration of PA reached 48.92 ± 2.06 g L^−1^, increased by 11.84%; the concentrations of SA and HAc were 10.36 ± 1.26 and 7.82 ± 0.92 g L^−1^, decreased by 35.53% and 34.06%, respectively. However, the total amount of the three organic acids was only 67.10 g L^−1^, decreased by 6.38%, implying an increase in other ineffective energy consumption.

**FIGURE 4:**
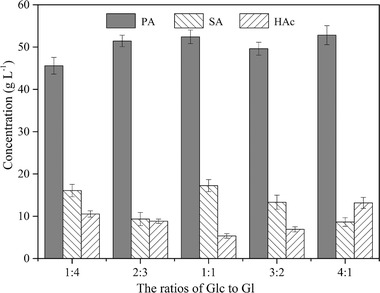
The concentrations of PA, SA, and HAc in *P. acidipropionici* fermentation process with different ratios of Glc to Gl as the mixed carbon source

Therefore, a combination of Glc and Gl as the carbon source was necessary for efficient co‐production of PA and SA. The optimum ratio of Glc to Gl was 1:1, and the resulting concentrations of PA, SA, and HAc were 52.42 ± 1.62, 17.24 ± 1.44, and 5.35 ± 0.53 g L^−1^, with productivity of 0.31, 0.10, and 0.03 g L^−1^ h^−1^, respectively (Figure 4). In particular, the productivity of PA increased by 19.84%, while the productivity of SA remained constant, compared with the fermentation process with Glc as the sole carbon source. Based on these findings, a Gl feeding procedure was established to further improve the fermentation efficiency. At the beginning, only 50 g L^−1^ of Glc were added and Gl was fed stepwise using the procedure described in Table 1. The uneven stepwise feeding procedure of Gl was beneficial for co‐production of PA and SA due to the non‐consistency of cell growth and metabolism. In the early stage of the fermentation process, sufficient carbon source can meet the needs of cell growth and metabolism; while in the latter stage of the fermentation process, the low concentration of carbon source can reduce the residual substrate and improve the substrate conversion rate. When Gl was fed using the “2‐1‐0.5″ procedure, the resulting concentrations of PA, SA, and HAc were 56.28 ± 1.76, 18.86 ± 1.03, and 3.74 ± 0.46 g L^−1^, with productivity of 0.34, 0.11, and 0.02 g L^−1^ h^−1^, respectively. The total amount of the three organic acids was 78.88 g L^−1^ (95.26% PA and SA).

**TABLE 1:** The Gl feeding procedure and its influence on the production of organic acids in the fed‐batch fermentation of *P. acidipropionici*

Gl feeding procedure^a^			PA			SA			HAc		
Stepwise concentration (g L^−1^)	Interval time (h)	Total amount (g L^−1^)	Concentration (g L^−1^)	Productivity (g L^−1^ h^−1^)	Yield^b^ (g g^−1^)	Concentration (g L^−1^)	Productivity (g L^−1^ h^−1^)	Yield^b^ (g g^−1^)	Concentration (g L^−1^)	Productivity (g L^−1^ h^−1^)	Yield^b^ (g g^−1^)
0.5	2	42	49.89 ± 1.62	0.297	0.542	21.85 ± 0.53	0.13	0.238	8.08 ± 0.46	0.048	0.088
1	4	42	49.87 ± 1.53	0.297	0.542	22.78 ± 0.75	0.136	0.248	6.39 ± 0.24	0.038	0.069
2	6	56	50.63 ± 1.91	0.301	0.478	16.09 ± 1.15	0.096	0.152	4.58 ± 0.61	0.027	0.043
2.5	8	52.5	52.62 ± 1.81	0.313	0.513	15.42 ± 0.93	0.092	0.150	4.75 ± 0.53	0.028	0.046
2‐1‐0.5^c^	4	47	56.28 ± 1.32	0.335	0.580	18.86 ± 1.03	0.112	0.194	3.74 ± 0.46	0.022	0.039

^a^ The Glc concentration in the medium was 50 g L^−1^.

^b^ The yield was calculated based on the total amount of Glc and Gl.

^c^ During the fermentation process, 2 g L^−1^ Gl was added every 4 h at 0–56 h; 1 g L^−1^ Gl was added every 4 h at 56–96 h; 0.5 g L^−1^ Gl was added every 4 h at 96–168 h.

### 3.3 Screening of ion‐exchange resin for PA removal

Seven anion exchange resins were screened at pH 6.5 to evaluate their applicability in the removal of PA from the fermentation broth. Characteristics of these resins including skeleton type, functional group, and particle size are shown in Table 2. Generally, the adsorption capacity of strong‐base resins is better than that of weak‐base resins, because of their ability to exchange with almost all acid radical anions. However, the adsorption capacity is not only related to the exchange capacity of the resin itself, but also to the adsorption conditions (e.g., the concentrations of various acid radicals in the fermentation broth). Therefore, the adsorption performance of the resins was mainly evaluated by their capacity to adsorb PA, because this compound causes feedback inhibition in the fermentation and removing it was the aim of using the exchange resin.

**TABLE 2:** The characteristics and adsorption properties of the screened resins

Resins	Skeleton	Functional group	Quality capacity (mmol g^−1^)	Volume capacity (mmol mL^−1^)	Particle size (mm)	Adsorption capacity in fermentation broth (pH 6.5) (mg g^−1^)		
						PA	HAc	SA
ZGA302	Styrene copolymer	‐NRCH_2_OH	≥3.6	≥1.4	0.45–1.20	42.38 ± 1.18	17.04 ± 1.75	50.66 ± 1.99
ZGA304	Styrene copolymer	‐N^+^(R)_3_	≥4.0	≥0.9	0.40–0.70	44.69 ± 2.02	20.55 ± 2.04	54.79 ± 2.31
ZGA351	Styrene copolymer	‐N^+^(R)_3_	≥3.8	≥1.2	0.50–0.80	47.32 ± 1.76	21.04 ± 1.91	58.85 ± 2.15
D213	Acrylic acid copolymer	‐N(CH_3_)_3_OH	≥3.5	≥0.8	0.40–0.70	45.71 ± 1.52	19.92 ± 1.35	56.43 ± 1.92
ZGA412	Acrylic acid copolymer	‐N(CH_3_)_2_	≥5.3	≥1.2	0.40–0.60	33.66 ± 1.38	17.33 ± 0.98	46.90 ± 1.56
ZGD630	Acrylic acid copolymer	‐N(CH_3_)_2_	≥7.0	≥2.0	0.40–1.25	54.34 ± 1.84	23.13 ± 2.10	65.76 ± 2.71
ZGA313	Acrylic acid copolymer	‐N(CH_3_)_2_	≥4.2	≥1.2	0.45–0.70	30.81 ± 1.05	17.95 ± 1.83	43.71 ± 1.89

The results showed that the adsorption capacity of PA was roughly equivalent among the four strong‐base resins, and ZGA351 showed slightly higher adsorption of PA, reaching 47.32 mg g^−1^. Among the three weak‐base resins, the adsorption capacity of ZGD630 was higher than that of the other resins, reaching 54.34 mg g^−1^. The mass exchange capacity of ZGD630 resin was ≥7 mM g^−1^, which was much higher than that of the above four strong‐base resins (range 3.5–4.0 mM g^−1^). Therefore, ZGD630 resin exhibited the highest PA adsorption in fermentation broth. In addition, the adsorption capacity of SA and HAc was also measured (Table 2). The results indicated that adsorption of SA from the fermentation broth was preferred to adsorption of PA and HAc; PA and HAc adsorbed on the resin would be replaced by SA as the volume of the fermentation broth increased or the amount of resin decreased, thereby reducing their adsorption.

### 3.4 Membrane separation coupled fermentation of *P. acidipropionici*

To relieve feedback inhibition by PA, a membrane separation coupled fermentation process was established. When the concentration of PA in the fermentation broth reached about 25–30 g L^−1^, *P. acidipropionici* cells were separated online using the multifunctional membrane device equipped with a 0.22‐μm spiral membrane module. As the small molecules (e.g., water, organic acids, and Glc) gradually permeated through the membrane module, the cell concentration in the fermenter gradually increased, up to fivefold. During the membrane separation process, some components penetrated the membrane pore, while some components adsorbed on the membrane surface or its inner pore walls non‐specifically, which led to a significant decrease of membrane flux at the beginning, with an average value of 75–85 L m^−2^ h^−1^. The cell activity was almost unchanged during the concentration process and cell survival was up to 95% in the fivefold concentrated suspension, due to the excellent biocompatibility of the process.

Membrane separation not only facilitated the pipeline connection of the entire coupled fermentation system, but also enabled the removal of PA by traditional fixed bed adsorption. The membrane concentrated penetrant can be loaded onto a pretreated ZGD630 resin chromatography column at a flow‐rate of 3–4 BV/h, consistent with the membrane filtration rate. As the penetrant flowed through the chromatography column, various acid radical ions were adsorbed, and the concentrations of SA, PA and HAc were decreased to 7.45, 9.23, and 2.96 g L^−1^, respectively. After adsorption, the flow‐through liquid was returned to the fermenter, and the acid radical ions were eluted from the resin with 1 M NaOH at a flow‐rate of 2–3 BV/h. During the adsorption process, the dynamic capacity of ZGD630 resin for PA was 72.32 mg g^−1^, increased by 33.09%. By analyzing the concentrations of Glc and amino‐nitrogen in the fermentation broth before and after adsorption, it was found that little of these components was adsorbed onto the ZGD630 resin, which was crucial for the establishment of the semi‐continuous fermentation process.

**FIGURE 5:**
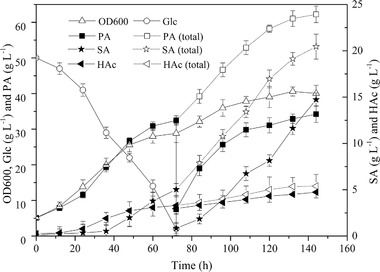
Time courses of OD600, Glc, PA, SA, and HAc in the *semi*‐continuous coupled fermentation process of *P. acidipropionici*

To establish the coupled fermentation process, the efficient integration of fermentation, membrane separation, and chromatographic adsorption must be realized. Comprehensive consideration of membrane flux, adsorption capacity, and sample loading rate should be undertaken; the 10‐L fermenter used at 70% capacity should be matched with membrane equipment with a 0.1–0.2 m^2^ membrane module and a chromatography column with bed volume of 2–2.5 L, so that the coupled operation can be completed within 30–40 min. Based on these parameters, semi‐continuous coupled fermentation of *P. acidipropionici* characterized by membrane separation and chromatography was performed. The results indicated that the removal of PA effectively promoted the metabolic synthesis of PA (Figure 5). In the traditional fed‐batch fermentation process, the productivity of PA decreased significantly (from 0.43 to 0.09 g L^−1^ h^−1^) when its concentration accumulated to about 25–30 g L^−1^, and the overall productivity was only 0.26 g L^−1^ h^−1^. After PA removal in the coupled fermentation process, its productivity remained almost unchanged. With the same carbon source consumption, the concentrations of PA and SA reached 62.22 ± 2.32 and 20.45 ± 1.34 g L^−1^, while the fermentation period was shortened from 168 to 144 h, corresponding to productivity of 0.43 and 0.14 g L^−1^ h^−1^, increased by 65.38% and 48.54%, respectively, calculated using the initial fermentation volume.

In the semi‐continuous coupled fermentation process with removal of PA, more substances and energy flowed in the direction of product synthesis, which effectively improved the fermentation efficiency and achieved co‐production of PA and SA. However, further detailed studies should be carried out with the aid of systems biology and synthetic biology to investigate the regulation of intracellular materials and energy metabolism in the coupled fermentation process. In addition, for the removal of PA, Wang et al. built an expanded bed adsorption device which allowed the cell‐containing fermentation broth to pass through directly [25]. However, expanded bed adsorption not only required rigorous operation (such as expansion coefficient), but the packing coefficient of the resin was also very low, which limited its application. With the popularization and application of membrane separation technology, its efficiency has gradually improved and the cost has been reduced. Introducing membrane separation into the fermentation system, coupled with traditional chromatography technology, can greatly improve the adsorption efficiency of PA. However, the adsorption conditions of the resin need to be further optimized. Around neutral pH (6.5–7.0), the adsorption capacity of ZGD630 resin was very low, and could not meet the requirements of efficient adsorption, resulting in the need for a large‐volume chromatography column (^1^/_3_–^1^/_2_ the volume of the fermenter). By adopting cation exchange resin and lowering the pH of the fermentation broth, one could significantly improve the adsorption capacity of the resin for PA, thereby effectively reducing the volume of the chromatography column and lowering production costs. These works are ongoing in our laboratory.

## 4 CONCLUDING REMARKS

Semi‐continuous coupled fermentation of *P. acidipropionici* was established to achieve co‐production of PA and SA. First, the effects of Glc and Gl on cell growth and product synthesis were analyzed. The optimal ratio of Glc to Gl as a combined carbon source was investigated, and the feeding procedure of Gl was optimized in fed‐batch fermentation. Then, ZGD630 anion exchange resin was screened for the removal of PA, and the efficient integration of fermentation, membrane separation, and chromatographic adsorption was investigated. Finally, semi‐continuous fermentation of *P. acidipropionici* was performed. The final productivity of PA and SA was 0.43 and 0.14 g L^−1^ h^−1^, increased by 65.38% and 48.54%, respectively. Therefore, membrane separation coupled fermentation of PA and SA can significantly improve the fermentation efficiency and has good prospects for industrial application.

## CONFLICT OF INTEREST

The authors declare that they have no known competing financial interests or personal relationships that could have appeared to influence the work reported in this paper.

## Data Availability

The data that support the findings of this study are available from the corresponding author upon reasonable request.
